# Commentary: Perceptions and needs of patients, caregivers and health professionals regarding an oncology community center: a qualitative study

**DOI:** 10.3389/fpubh.2025.1682149

**Published:** 2025-11-12

**Authors:** Shuoyang Xu, Jing Wang, Wei Zhao, Wenjiang Wu

**Affiliations:** Shenzhen Hospital (Futian) of Guangzhou University of Chinese Medicine, Shenzhen, Guangdong, China

**Keywords:** oncology community centers, cancer care, general practitioners (GPs), caregivers, cancer patients

## Introduction

Mitnik et al.'s (1) timely study illuminates the human dimension of decentralizing cancer care through Oncology Community Centers (OCCs). Their qualitative exploration reveals a core tension: the clash between the undeniable benefits of geographical accessibility and the deep-seated psychological need for clinical security? associated with large hospitals. As healthcare systems globally grapple with rural-urban disparities, this work exposes critical undercurrents—the *proximity-security paradox* and the untapped potential of *General Practitioners (GPs)*—that demand deeper consideration in OCC implementation. While Mitnik et al. first identified this tension, our commentary systematically conceptualizes it as the “proximity-security paradox” and draws practical implications for design and policy. This commentary aims to: first, explore and conceptualize the “proximity-security paradox” as a framework capturing the tension identified by Mitnik et al.; and second, discuss potential multi-level strategies—addressing both systemic integration and interpersonal dynamics—to resolve this paradox. This commentary extends the original study by not only conceptualizing the problem but also offering a synthesis for implementation, while highlighting systemic barriers (e.g., GP reimbursement models) critical for the sustainable success of OCCs. Moreover, we emphasize that such systemic strategies must be coupled with attention to interpersonal dynamics—such as trust-building and environmental familiarity—which are equally critical to resolving the paradox.

## The proximity-security paradox: beyond geography

### Conceptualizing the proximity-security paradox

The study powerfully captures the patient dilemma: while welcoming reduced travel burdens (“leave the house without 3.5 h travel”), participants expressed profound anxiety about losing the perceived safety net of the hospital's “complete staff.” This anxiety transcends logistics, tapping into a profound state of existential vulnerability well-documented in psycho-oncology (2). While Lo et al. focused on managing this vulnerability within the hospital setting, our commentary extends this by examining how decentralizing care inherently exacerbates this anxiety. This aligns with global patterns where patients prioritize perceived credibility over convenience (3), underscoring that trust requires deliberate design features within the OCC model, and cannot be assumed.

To understand the mechanisms behind this trust deficit, we consider the cognitive biases underlying this trust deficit: patients' heuristic reliance on hospital size as a proxy for safety, a phenomenon noted in healthcare decision-making literature (4), which is rooted in systemic fragmentation and lack of transparent outcome data. To address this trust deficit mechanistically, we suggest a potential intervention: the integration of real-time outcome dashboards at OCCs. We hypothesize that this could provide transparent quality metrics, thereby shifting patient focus from institutional size to evidence-based security. To clinically translate this concept, a pilot study could be designed to implement and evaluate patient education modules based on this dashboard data, with the goal of enhancing adherence by reducing existential anxiety. While Mitnik's hospital-affiliated OCC model represents a significant step forward, challenges to ensuring relational continuity remain.

### Practical strategies for OCC design to mitigate the paradox

Future OCC frameworks must therefore proactively address this paradox through integrated, multi-level strategies, prioritizing the human and environmental foundations of trust:

#### Strengthening relational and environmental continuity

First and foremost, deploying dedicated patient navigators specifically trained to address emotional anxieties and bridge logistical gaps is crucial. This interpersonal security must be complemented by a consciously designed “homely” environment (as detailed in a later section), which uses domestic-scale furniture, warm lighting, and sound-absorbing materials to reduce stress and foster open communication. This foundation of interpersonal and environmental trust makes subsequent technological solutions more acceptable.

#### Leveraging technology

Building upon this foundation, integrating real-time telehealth hubs that link OCCs directly to hospital tumor boards for instant specialist consultation.

#### Building preparedness

Further reinforcing security, implementing mandatory simulated emergency drills involving OCC staff and local EMS to enhance procedural confidence and coordination.

These strategies form an integrated model that leverages hospital resources while embedding community access and primary care, aiming to balance the proximity-security continuum.

This conceptual approach operationalizes broader calls for innovative frameworks (5) by providing a specific, evaluable model for designing decentralized care based on the measurable dimensions of proximity and security.

## GP integration: from peripheral to central players

Beyond technological solutions, the integration of General Practitioners (GPs) presents a critical human-centric strategy to resolve the proximity-security paradox, by leveraging their unique position within the community to build trust and provide continuous care.

### GP assets and current underuse

Mitnik notes GPs' expressed willingness to collaborate (“direct communication, not referrals”), yet patients dismissed their relevance beyond comorbidities, reflecting systemic fragmentation and a missed opportunity. GPs possess unique longitudinal relationships and contextual understanding of patients' lives—assets critically underutilized in traditional oncology pathways. To activate this potential, OCCs need to clearly delineate GP roles within a tiered shared-care model (e.g., oncologists managing acute-phase treatment while GPs lead survivorship care and comorbidity management). This requires defining standardized protocols, leveraging digital health tools for enhanced care coordination, and providing systematic oncology-specific training and support to build GP competence and confidence. Evidence suggests that cancer patients value GP involvement in coordinated care models (6, 12), yet their role remains underutilized. Piloting such a model in phases—starting with comorbidity management during active treatment and scaling to full survivorship care—could measure reductions in hospital readmissions as an early outcome, thereby demonstrating its value in mitigating systemic fragmentation.

### Structural enablers, barriers, and international lessons

The underutilization of GPs is not incidental but rooted in structural barriers, primarily reimbursement models that do not compensate for care coordination and training gaps in specialist cancer care. However, evidence from other systems offers clear solutions. Australia's success with blended payment schemes demonstrates a viable model to financially incentivize GPs' coordinated care roles, directly addressing the remuneration barrier (7, 8). Concurrently, Canada's Project ECHO provides a tele-mentoring framework that effectively overcomes training and isolation barriers by connecting community-based GPs to specialist networks for case-based learning (9). These two models provide a complementary blueprint for OCCs: one addressing economic incentives, the other building clinical confidence and community. Piloting such initiatives within OCCs could generate empirical evidence to inform policymakers.

By actively integrating GPs through such models, OCCs can effectively extend the security of the hospital ecosystem into the community, directly mitigating patients' existential anxiety by providing a trusted, familiar, and competent point of care close to home. GP integration uniquely resolves the proximity-security paradox by providing a trusted human anchor that complements technological solutions, bridging the gap between institutional expertise and community accessibility.

## The “homely” environment as a theoretical component of security

Crucially, the systemic integrations we propose must be designed to augment, not replace, the supportive, “homely” interpersonal environment emphasized by Mitnik et al. This element should be theorized not as a mere aesthetic feature but as a fundamental contributor to relational continuity and perceived safety, a link supported by evidence in healthcare design literature (10, 11). A welcoming and familiar environment directly mitigates existential anxiety by fostering trust and reducing the institutional alienation often associated with large hospitals. For instance, evidence suggests that features such as domestic-scale furniture, access to natural light, and artwork can significantly lower physiological markers of stress and anxiety in patients (10).

The mechanism through which this operates involves the reduction of unfamiliarity and the promotion of relational cues. A “homely” environment mitigates the power imbalance and intimidation often felt in sterile clinical settings by fostering familiarity—through comfortable seating arrangements that facilitate open conversation, warm lighting, and personal touches. This subconsciously signals to patients that they are in a caring, person-centered space, thereby lowering defensive anxiety and building the foundational trust necessary for effective care communication and adherence. This interpersonal security is the indispensable counterpart to technological and procedural security; together, they form the dual pillars for resolving the proximity-security paradox.

**Figure 1:**
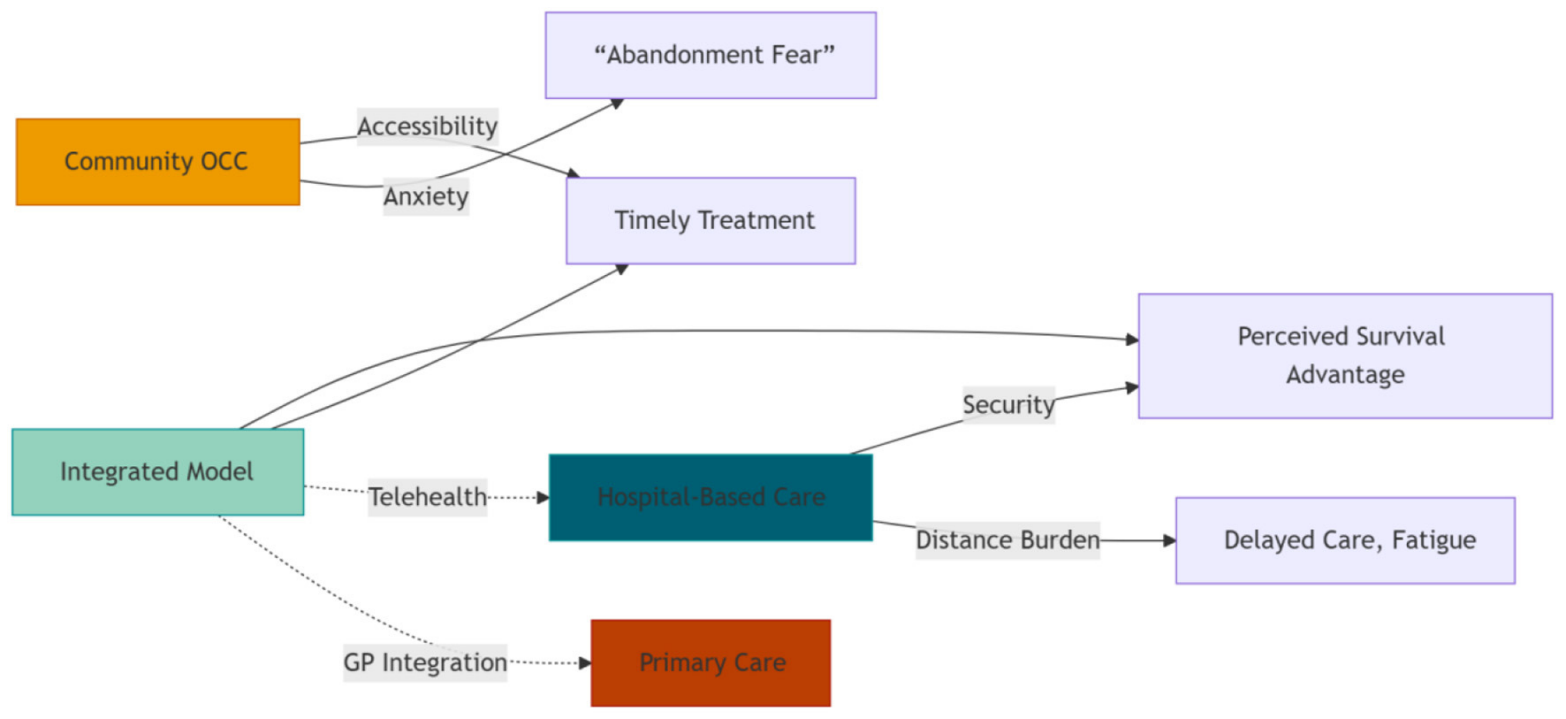
Balancing the Proximity-Security Continuum: An integrated model leverages hospital resources while embedding community access and primary care.

Therefore, OCC training programs and design principles ought to prioritize empathic communication and environmental comfort with the same rigor as technical proficiency. This could be operationalized through: (1) mandatory communication simulation training for staff, focusing on active listening and anxiety de-escalation techniques in a community setting; and (2) the adoption of minimum design standards for OCCs, developed through patient co-design workshops, which incorporate elements such as sound-absorbing materials for privacy, adjustable lighting, and residential-style furniture to create a physically and psychologically comfortable environment. Our proposed model (Figure 1) visualizes this necessary integration, positioning the “homely” OCC environment as the foundational layer upon which both proximity and technological security are built, ensuring that accessibility does not come at the cost of patient-centeredness.

## Discussion

Building upon the foundational qualitative insights of Mitnik et al. (1), this commentary offers a novel conceptual and practical extension by introducing the “proximity-security paradox” as a defining framework. Our proposed “security-accessibility matrix” serves as a conceptual heuristic to guide decision-making by framing OCC design along two axes: proximity and security.

At the clinical level, this matrix translates into care models that must strategically balance local access points (e.g., OCCs) with robust security layers (e.g., virtual specialist networks). A key operational challenge involves fostering trust and standardized practices across these diverse settings.

At the policy level, validating and scaling such integrated models requires concomitant policy reforms. Future research must quantify the impact on hard outcomes like hospitalizations and cost-efficiency. This evidence is essential to inform critical policy changes, particularly in GP reimbursement models and funding for telehealth infrastructure, which are the true enablers for sustainable OCC implementation.

In summary, whereas Mitnik et al. expertly documented stakeholder perceptions, our commentary provides a synthesizing framework and a forward-looking roadmap. We argue that resolving the proximity-security paradox is a sociocultural challenge requiring deliberate strategies to engineer trust, activate primary care networks, and design nurturing environments. We therefore recommend: to pilot and evaluate these integrated models within the context of national cancer control plans and global health security frameworks, such as those advocated by the WHO, to ensure that the decentralization of cancer care achieves its goal of equitable, accessible, and truly patient-centered security for all.
